# Prevalence and Frequency of mHealth and eHealth Use Among US and UK Smokers and Differences by Motivation to Quit

**DOI:** 10.2196/jmir.4420

**Published:** 2015-07-04

**Authors:** Belinda Borrelli, Yvonne Kiera Bartlett, Erin Tooley, Christopher J Armitage, Alison Wearden

**Affiliations:** ^1^ Boston University Henry M Goldman School of Dental Medicine Boston, MA United States; ^2^ Manchester Centre for Health Psychology School of Psychological Sciences, Manchester Academic Health Science Centre University of Manchester Manchester United Kingdom; ^3^ Feinstein College of Arts & Sciences Roger Williams University Bristol, RI United States

**Keywords:** smoking cessation, eHealth, mHealth, health behavior, motivation, text messaging

## Abstract

**Background:**

Both mHealth and eHealth interventions for smoking cessation are rapidly being developed and tested. There are no data on use of mHealth and eHealth technologies by smokers in general or by smokers who are not motivated to quit smoking.

**Objective:**

The aims of our study were to (1) assess technology use (eg, texting, social media, Internet) among smokers in the United States and United Kingdom, (2) examine whether technology use differs between smokers who are motivated to quit and smokers who are not motivated to quit, (3) examine previous use of technology to assist with smoking cessation, and (4) examine future intentions to use technology to assist with smoking cessation.

**Methods:**

Participants were 1000 adult smokers (54.90%, 549/1000 female; mean age 43.9, SD 15.5 years; US: n=500, UK: n=500) who were recruited via online representative sampling strategies. Data were collected online and included demographics, smoking history, and frequency and patterns of technology use.

**Results:**

Among smokers in general, there was a high prevalence of mobile and smartphone ownership, sending and receiving texts, downloading and using apps, using Facebook, and visiting health-related websites. Smokers who were unmotivated to quit were significantly less likely to own a smartphone or handheld device that connects to the Internet than smokers motivated to quit. There was a significantly lower prevalence of sending text messages among US smokers unmotivated to quit (78.2%, 179/229) versus smokers motivated to quit (95.0%, 229/241), but no significant differences between the UK groups (motivated: 96.4%, 239/248; unmotivated: 94.9%, 223/235). Smokers unmotivated to quit in both countries were significantly less likely to use a handheld device to read email, play games, browse the Web, or visit health-related websites versus smokers motivated to quit. US smokers had a high prevalence of app downloads regardless of motivation to quit, but UK smokers who were motivated to quit had greater prevalence of app downloads than smokers unmotivated to quit. US smokers were significantly more likely to have a Facebook account (87.0%, 435/500) than UK smokers (76.4%, 382/500), but smokers unmotivated to quit in both countries used Facebook less frequently than smokers motivated to quit. Smokers who were unmotivated to quit were less likely to have used eHealth or mHealth platforms to help them quit smoking in the past and less likely to say that they would use them for smoking cessation in the future.

**Conclusions:**

Although smokers unmotivated to quit make less use of technology than smokers motivated to quit, there is sufficient prevalence to make it worthwhile to develop eHealth and mHealth interventions to encourage cessation. Short and low-effort communications, such as text messaging, might be better for smokers who are less motivated to quit. Multiple channels may be required to reach unmotivated smokers.

## Introduction

The current prevalence of cigarette smoking is 18.1% in the United States [1] and 19% in the United Kingdom [2], with substantially higher prevalence among specific underserved subpopulations [3,4]. Although rates of smoking have declined over the last 10 years, this decline has recently plateaued [1,2], possibly due to lower cessation among specific subpopulations, such as those with low income and education and those with medical and psychiatric comorbidities [4]. In the United States and United Kingdom, national studies have shown that 29% to 31% of smokers are not interested in quitting smoking in either the short or long term [5,6].

The use of technology-based interventions, such as those delivered through Internet (eHealth) and mobile phones (mHealth), may enhance the reach of smoking cessation interventions given the lack of disparities by race, education, and income in use of these technologies [7-10]. For example, rates of mobile phone penetration are expected to reach close to 100% [11]; 81% of mobile phone owners use their phone to send or receive text messages [12] and the average number of text messages per day is high across all racial and ethnic groups (black: mean 70.1, median 20; Hispanic: mean 48.9, median 20; white: mean 31.2, median 10 [8]). Moreover, those who are Latino, black, or aged between 18 and 49 years are more likely to gather health information through their mobile phones [7].

Both mHealth and eHealth interventions have been shown to be effective for smoking cessation among those who are ready to quit [13-16]. However, mHealth and eHealth have not yet been used to motivate quit attempts in smokers who are not motivated to quit. Given the extent to which technology has become integrated into people’s everyday lives, targeting smokers who are not motivated to quit through these platforms may help to jump-start stalled smoking cessation rates. To our knowledge, there are no studies that assess mHealth and eHealth use (and frequency of use) by smokers in general or by smokers who are not motivated to quit smoking. Before the development of mHealth and eHealth interventions for these smokers, it is necessary to ascertain their level of engagement with technology. This information could help intervention planners and funders to find out where the smokers “hang out” and direct resources accordingly. Thus, the aims of our study are to (1) assess technology use (eg, texting, social media, Internet) among smokers in the United States and United Kingdom, (2) examine whether use of technology differs between smokers who are motivated to quit and smokers who are not motivated to quit, (3) examine previous use of technology to assist with smoking cessation, and (4) assess future intentions to use technology to assist with smoking cessation.

We assessed smokers in the United States and United Kingdom because they are the 2 English-speaking markets with the most active users of iOS (iPhone/iPad) and Android devices [17]. Given that smokers who are not ready to quit comprise a large minority of the smoking population, understanding their use of, and level of engagement with, technology could help expand the reach of current smoking cessation interventions and develop interventions specifically targeted to, and tailored for, this population.

## Methods

### Participants

Participants were 1000 current smokers: 500 in the United States and 500 in the United Kingdom. In each country, we recruited 250 smokers who did not want to quit smoking (defined as “does not plan to quit smoking cigarettes in the next 30 days”) and 250 smokers who were ready to quit smoking within 30 days and were either (1) currently investigating options for help with quitting smoking or (2) had set a quit date within 30 days. Participants were eligible if they were current, regular smokers (ie, smoke at least 3 tobacco cigarettes per day for the past year and smoked more than 100 cigarettes in their lifetime) and aged 18 years or older.

A total of 1767 people completed the initial screening questions; 572 were screened out because they did not meet eligibility criteria, 32 were removed due to random responding (see Data Analyses), and 89 were removed to ensure that the sample was representative of age and gender. Of those who were eligible to participate (n*=*1074), one participant was not able to be categorized as “motivated to quit” or “unmotivated to quit” and 6.80% (73/1074) did not complete the survey. Thus, the final sample was 1000 smokers: 500 in the United States and 500 in the United Kingdom.

### Procedure

Participants were recruited through online survey sampling conducted by Toluna, Inc. Toluna has processes in place to ensure that respondents do not misrepresent themselves to gain access to a study for which they are not eligible and that no participant takes part in any study more than once. Participants were recruited from Toluna’s panel, Toluna-affiliated partnerships, websites, and social media. All potential participants were extensively verified and underwent checks to ascertain their identity and location. Toluna also checked for duplication within the panel before permitting access to the survey. Participants received 4000 “panel points” for survey completion. These points could be redeemed for vouchers for shops and services, redeemed as cash, or used to enter prize drawings at the participant’s discretion.

All data were collected during one week in August 2014 and 21.90% (219/1000) of the sample completed the survey on their mobile phone. Toluna removed all identifiable information before transferring the dataset to investigators (ie, removal of IP addresses). Toluna adheres to and exceeds various data security protocols regarding personal identifiable information for its panelists and its research respondents and they meet all international data security protocols (eg, ISO27001). Ethical approval was obtained from The Miriam Hospital in the United States and the University of Manchester in the United Kingdom, and informed consent was obtained from participants before participation.

### Measures

Demographics and smoking history were assessed with age, gender, marital status, ethnicity, employment, years of education, and number of cigarettes smoked per day. We assessed nicotine dependence with one item from the Fagerström Test for Nicotine Dependence (FTND) [18]: “Do you smoke within 30 minutes of waking? (yes/no).” This single item is highly correlated with the full scale [19]. General use of technology was assessed with the Technology Use Questionnaire, a series of questions developed for this study regarding use and frequency of use of different types of Internet and mobile technologies. We also used the social media and smartphone usage subscales from the Media and Technology Usage and Attitudes Scale [20]. We defined “regular users” as those who used a feature several times per month or once per day or more. The Technology-Assisted Smoking Cessation Questionnaire, also developed for this study, was used to assess the use of technology for smoking cessation. It consisted of 5 items asking about previous use and 5 items asking about future intentions to use each of the following technologies for smoking cessation: the Internet, text messages, mobile phone apps, Twitter, or Facebook.

### Data Analysis

Data were cleaned before analyses. We eliminated (1) straight-liners (n=8), defined as respondents who selected the same answer option for all items within a scale so that they completed the survey as quick as possible with minimum effort and (2) speeders (n=24), who did not carefully read the questions and provided random responses as evidenced by completing the survey more quickly than the typical respondent (ie, completing the survey in less than half the median time). Toluna also checked for respondents who filled in random letters into open-ended question fields, those who inserted offensive words, and duplicate survey takers, but none were noted in our sample. The final sample size was 1000.

We analyzed the overall prevalence of technology use and frequency of use by smokers, as well as compared differences between smokers who were motivated to quit and smokers who were not motivated to quit. Independent group *t* tests were computed for continuous variables and chi-square tests were computed for categorical data. For chi-square tests, standardized residuals were calculated and values equal to or greater than 1.96 or equal to or less than -1.96 (the critical value that corresponds to α<.05) were considered large (ie, [21]). We used hierarchical logistic regression to ascertain whether motivation group (motivated to quit vs unmotivated to quit) was related to device ownership and technology use after controlling for demographics (age, education, ethnicity, and income).

## Results

### Sample Demographics

Only 6.80% (73/1074) of the sample did not complete the survey. Noncompleters (n=73) were significantly more likely to be female (χ^2^_1_=6.2, *P=*.01) and were more likely to complete the survey on a mobile device (χ^2^_1_=7.3, *P*<.007). There were no differences between completers and noncompleters in age, country, group (motivated vs unmotivated), number of cigarettes smoked per day, when they planned to quit smoking, and confidence in their ability to quit smoking.

The sample was comprised of 54.90% (549/1000) female smokers (Table 1) and the ethnic composition was 82.60% (826/1000) white, 6.50% (65/1000) black, 3.70% (37/1000) Hispanic/white, 0.40% (4/1000) Hispanic/black, 3.10% (31/1000) Asian, 0.40% (4/1000) American Indian/Alaskan Native, 0.30% (3/1000) Native Hawaiian or Pacific islander, 1.50% (15/1000) multiracial, and 1.50% (15/1000/) prefer not to say. Slightly more than half (55.90%, 559/1000) of the sample was employed (full- or part-time) and 61.30% (613/1000) were partnered (ie, married, engaged, living together, or in relationship but not living together). Participants smoked mean 16.5 (SD 13.4) cigarettes per day and 72.30% (723/1000) smoked within 30 minutes of waking, suggesting a high level of behavioral dependence on nicotine [19].

We assessed demographic differences between smokers who were motivated to quit and smokers who were not motivated to quit. Smokers who were not motivated to quit were significantly older (*t*_998_=14.31, *P*<.001) and less likely to be employed (χ^2^_1_=19.3, *P*<.001) than smokers who were motivated to quit. Ethnic minorities were more likely to be motivated to quit (71.7%, 114/159) than white smokers (45.5%, 376/826; χ^2^_1_=36.6, *P*<.001). There were no other significant demographic differences between motivated and unmotivated smokers (Table 1). In terms of differences between the samples in the 2 countries, the US sample had a significantly higher proportion of females (χ^2^_1_=7.5, *P*=.006), were less likely to report paid employment (χ^2^_1_=7.5, *P*=.006), and were significantly more likely to complete the survey on a mobile phone (26.8%, 134/500) than UK participants (17.0%, 85/500; χ^2^_1_=14.0, *P*<.001).

**Table 1:** Demographics of the total sample and by motivation to quit.

Variable	Total sample N=1000	Smokers not motivated to quit n=500	Smokers motivated to quit n=500	χ^2^_1_	*t*_998_	*P*
Female, n (%)	549 (54.90)	286 (52.1)	263(47.9)	2.1		.14
Age (years), mean (SD)	43.9 (15.4)	50.3 (14.1)	37.5 (14.1)		14.31	<.001
Ethnicity (white), n (%)	826 (82.60)	450 (54.5%)	376 (45.5)	36.6		<.001
<University education,^a^ n (%)	488 (49.10)	253 (51.8)	235 (48.2)	1.10		.30
Employed full- or part-time, n (%)	559 (55.90)	245 (43.8)	314 (56.2)	19.3		<.001
Partnered/in relationship, n (%)	613 (61.30)	312 (50.9)	301 (49.1)	0.5		.48
Cigarettes smoked/day, mean (SD)	16.6 (13.4)	17.3 (12.6)	15.8 (14.1)		1.76	.08

^a^ Participants selecting “I don’t know” were counted as missing.

### Smokers in the United States

#### Prevalence of Handheld Device Ownership and Differences by Motivation to Quit

Of the US sample, 92.8% (464/500) reported owning a mobile phone and 75.9% (352/464) of these were smartphones (Table 2). In addition, 79.6% (374/470) reported that they owned a handheld device that connects to the Internet and 49.8% (249/500) reported owning a tablet. Smokers who were motivated to quit were significantly more likely to own a mobile device, such as a mobile phone or tablet (95.6%, 239/250), than smokers who were not motivated to quit (90.0%, 225/250). Of those who owned devices, smokers who were motivated to quit (86.3%, 208/500) were more likely to be able to access the Internet on their mobile phone or tablet versus smokers not motivated to quit (72.5%, 166/500). There were no differences between groups after controlling for demographics in regression analyses.

#### Prevalence of Types of Technology and Frequency of Use

Of those who had devices capable of text messaging, only 13.2% (62/470) reported that they never send text messages and 10.6% (50/470) reported that they never receive text messages. Of those who sent text messages, 32.8% (134/408) sent 2 to 9 texts per day and 34.1% (139/408) sent 10 or more texts per day (“supertexters”). Of those who received text messages, 33.5% (141/421) received 2 to 9 texts per day and 32.8% (138/421) received 10 or more texts per day.

Of those who reported having handheld devices (94.0% 470/500), the most common features regularly used (ie, several times per month or more) were reading email (68.3%, 321/470), browsing the Web (70%, 329/470), taking photos (66.0%, 310/470), using apps (66.6%, 313/470), and playing games (58.7%, 276/470). Additionally, 70% (350/500) of this sample had visited health-related websites on either a handheld device or computer; of these, only 34.0% (119/350) visited them regularly (twice per week or more). Of those who owned a handheld device capable of accessing the Internet, 91.4% (342/374) reported that they had previously downloaded an app; of those, 26.4% (n95/360) said that they used it for 1 month or more (but less than 1 year) and 34.4% (124/360) said that they used it for 1 year or more. Only 10.0% (36/360) reported that they downloaded an app, but used it for less than 1 day. For Facebook, 87% (435/500) reported having a Facebook account; 20.5% (89/435) checked it once per day and 49.7% (n216/435) checked it more than once per day.

#### Prevalence of Types of Technology and Frequency of Use: Differences by Motivation to Quit

There were differences in technology use and frequency of use between smokers who were motivated to quit and smokers who were not motivated to quit. Only 5.0% (12/241) of smokers who were motivated to quit reported never sending text messages versus 21.8% (50/229) of smokers who were not motivated to quit. Differences between motivation groups were maintained after demographics were controlled for in regression analysis (*P=*.01, Wald=6.08, SE*=*0.40). Of those who sent texts, there was a significant relationship between type of smoker (motivated vs unmotivated) and frequency of texting. Examination of the standardized residuals suggested that motivated smokers were more likely to be supertexters (texting >10 times per day).

Smokers who were motivated to quit were significantly more likely than smokers unmotivated to quit to regularly use their handheld devices to accomplish a variety of tasks (eg, email, browse the Web, use apps, play games; Table 2). Of smokers who were not motivated to quit, 43.2% (108/250) reported that they never visited websites related to health issues versus 16.8% (42/250) of smokers who were motivated to quit (differences that were maintained after controlling for demographics; *P*=.006, Wald=7.51, SE=0.25). Of those who visited health-related websites, smokers who were motivated to quit visited these websites more frequently than smokers unmotivated to quit.

The majority of smokers who were motivated to quit (93.8%, 195/208) reported that they previously downloaded an app and this prevalence was not significantly different from that of unmotivated smokers (88.6%, 147/166; *P*=.07). There were no significant differences between motivation groups in the longest length of time of app use. Although there were no significant differences between motivated and unmotivated smokers in the prevalence of having a Facebook account (*P*=.14), smokers who were motivated to quit checked Facebook more frequently (>once per day: 57.8%, 129/223) than smokers who were unmotivated to quit (>once per day: 41.0%, 216/435).

**Table 2:** Prevalence of technology use among smokers in the United States and differences in technology use by motivation to quit.

Variable			Total US sample, n (%) n=500	Smokers not motivated to quit, n (%) n=250	Smokers motivated to quit, n (%) n=250	χ^2^ (*df*)	*P*
**Device ownership**							
	Own a mobile		464 (92.8)	225 (90.0)	239 (95.6)	5.9 (1)	.02
	Own a tablet		249 (49.8)	100 (40.0)	149 (59.6)	19.2 (1)	<.001
	Mobile is a smartphone		352 (75.9)	153 (68.0)	199 (83.3)	14.7 (1)	<.001
	Internet-enabled handheld device		374 (79.6)	166 (72.5)	208 (86.3)	13.8 (1)	<.001
**Texting**							
	Send text messages		408 (86.8)	179 (78.2)	229 (95.0)	29.1 (1)	<.001
	Receive text messages		421 (89.6)	190 (83.0)	231 (95.9)	20.9 (1)	<.001
	**Frequency of sending texts** ^a^					33.4 (5)	<.001
		≤1 texts per month	31 (7.6)	14 (7.8)	17 (7.4)		
		2-4 texts per month^b^	29 (7.1)	22 (12.3)	7 (3.1)		
		2-6 texts per week^c^	56 (13.7)	35 (19.6)	21 (9.2)		
		1 text per day	19 (4.7)	10 (5.6)	9 (3.9)		
		2-9 texts per day	134 (32.8)	58 (32.4)	76 (33.2)		
		≥10 texts per day^b^	139 (34.1)	40 (22.3)	99 (43.2)		
	**Frequency of receiving texts** ^d^					32.4 (5)	<.001
		≤1texts per month	34 (8.1)	17 (8.9)	17 (7.4)		
		2-4 texts per month^c^	36 (8.6)	26 (13.7)	10 (4.3)		
		2-6 texts per week^c^	55 (13.1)	36 (18.9)	19 (8.2)		
		1 text per day	17 (4.0)	8 (4.2)	9 (3.9)		
		2-9 texts per day	141 (33.5)	61 (32.1)	80 (34.6)		
		≥10 texts per day^b^	138 (32.8)	42 (22.1)	96 (41.6)		
**Features of a handheld device used regularly**							
	Read email		321 (68.3)	137 (59.8)	184 (76.3)	14.8 (1)	<.001
	Get directions or use navigation (eg, GPS)		226 (48.1)	83 (36.2)	143 (59.3)	25.1 (1)	<.001
	Browse the Web		329 (70.0)	136 (59.4)	193 (80.1)	24.0 (1)	<.001
	Listen to music		276 (58.7)	103 (45.0)	173 (71.8)	34.8 (1)	<.001
	Take photos		310 (66.0)	121 (52.8)	189 (78.4)	34.2 (1)	<.001
	Check the news		270 (57.4)	106 (46.3)	164 (68.0)	22.7 (1)	<.001
	Record video		168 (35.7)	57 (24.9)	111 (46.1)	22.9 (1)	<.001
	Use apps (for any purpose)		313 (66.6)	130 (56.8)	183 (75.9)	19.4 (1)	<.001
	Search for information		315 (67.0)	127 (55.5)	188 (78.0)	27.0 (1)	<.001
	Play games by yourself		276 (58.7)	114 (49.8)	162 (67.2)	14.7 (1)	<.001
	Play games with other people		168 (35.7)	46 (20.1)	122 (50.6)	47.7 (1)	<.001
**Apps**							
	Previous app download^e^		342 (91.4)	147 (88.6)	195 (93.8)	3.2 (1)	.07
	Longest used app for^e^					7.1 (4)	.13
		<1 day	36 (10.0)	13 (8.6)	23 (11.1)		
		≥1 day but <1 week	56 (15.6)	21 (13.8)	35 (16.8)		
		≥1 week but <1 month	49 (13.6)	16 (10.5)	33 (15.9)		
		≥1 month but <1 year	95 (26.4)	50 (32.9)	45 (21.6)		
		≥1 year	124 (34.4)	52 (34.2)	72 (34.6)		
**Facebook**							
	Facebook account		435 (87.0)	212 (84.8)	223 (89.2)	2.1 (1)	.14
	**Frequency of checking** ^f^					15.7 (5)	.008
		Never	8 (1.8)	3 (1.4)	5 (2.2)		
		≤Once per month	24 (5.5)	17 (8.0)	7 (3.1)		
		2-4 times per month	26 (6.0)	15 (7.1)	11 (4.9)		
		2-6 times per week	72 (16.6)	42 (19.8)	30 (13.5)		
		Once per day	89 (20.5)	48 (22.6)	41 (18.4)		
		>Once per day	216 (49.7)	87 (41.0)	129 (57.8)		
**Visit health-related websites**			350 (70.0)	142 (56.8)	208 (83.2)	41.5 (1)	<.001
	**Frequency of visits** ^g^					38.5 (4)	<.001
		≤Once per month^b^	125 (35.7)	71 (50.0)	54 (26.0)		
		2-4 times per month	106 (30.3)	45 (31.7)	61 (29.3)		
		2-6 times per week	54 (15.4)	18 (12.7)	36 (17.3)		
		Once per day	34 (9.7)	7 (4.9)	27 (13.0)		
		>Once per day^b^	31 (8.9)	1 (0.7)	30 (14.4)		

^a^ Of those who sent text messages (n*=*408).

^b^ Standardized residual ≥2.58 or ≤–2.58.

^c^ Standardized residual ≥1.96 or ≤–1.96

^d^ Of those who receive text messages (n*=*421).

^e^ Of those who can access the Internet on their handheld device (n*=*374).

^f^ Of those who have a Facebook account (n*=*435).

^g^ Of those who visit websites related to health issues (n*=*350).

### Smokers in the United Kingdom

#### Prevalence of Handheld Device Ownership and Differences by Motivation to Quit

Of the UK sample, 95.8% (479/500) reported owning a mobile phone and 82.9% (397/479) of these were smartphones (Table 3). In all, 85.7% (414/483) reported that they owned a handheld device that connects to the Internet and 56.2% (281/500) reported owning a tablet. Smokers who were motivated to quit were significantly more likely to own a handheld device (mobile or tablet) than smokers unmotivated to quit (99.2%, 248/250 vs 94.0%, 235/250). Of those who owned handheld devices, smokers who were motivated to quit (90.7%, 225/248) were significantly more likely to have a device that connects to the Internet than smokers who were not motivated to quit (80.4%, 189/235). There were no differences between motivation groups after controlling for demographics (age, education, and income) in logistic regressions.

#### Prevalence of Types of Technology and Frequency of Use Among UK Smokers

The vast majority of UK smokers reported that they send (95.7%, 462/483) and receive (97.7%, 472/483) text messages. Of those who send text messages, 36.1% (167/462) send 2 to 9 texts per day and 21.4% (99/462) were supertexters. Of those who received text messages, 36.7% (173/472) received 2 to 9 texts per day and 21.2% (100/472) received 10 or more texts per day.

Of those who had handheld devices (96.6%, 483/500), the most common features regularly used (ie, several times per month or more) were browsing the Web (70.2%, 339/483), reading email (70.0%, 338/483), searching for information (68.9%, 333/483), using apps (66.5%, 321/483), and taking photos (62.1%, 300/483). Of those who ever visited health-related websites (61.0%, 305/500), 24.9% (76/305) visited them regularly (twice per week or more). Of those with Internet-enabled handheld devices (85.7%, 414/483), 86.2% (357/414) reported that they had previously downloaded an app; 26.6% (95/357) said that they used it for 1 month or more (but less than 1 year) and 34.2% (122/357) said that they used it for 1 year or more. Only 10.1% (36/357) said that they downloaded an app but used it less than 1 day; 29.1% (104/357) used it more than 1 day but less than 1 month. Of the UK sample, 76.4% (382/500) reported having a Facebook account; 20.7% (79/382) checked it once per day and 53.7% (205/382) checked it more than once per day.

#### Prevalence of Types of Technology and Frequency of Use: Differences by Motivation to Quit

There were no significant differences between smokers who were motivated to quit and smokers who were not motivated to quit in whether or not they texted; however, the prevalence of texting was very high among both groups: only 3.6% (9/248) of smokers who were motivated to quit reported never sending text messages versus 5.1% (12/235) of smokers who were not motivated to quit (*P=*.43). However, of those who texted, there was a significant relationship between motivation to quit and frequency of texting. Examination of the standardized residuals indicated that smokers who were not motivated to quit were less likely to reside in the supertexter category (eg, >10 texts per day). Smokers who were motivated to quit were significantly more likely to regularly use their handheld devices for a variety of tasks (eg, email, browse the Web, use apps) than smokers who were not motivated to quit (Table 3). Of smokers who were not motivated to quit, 54.8% (137/250) reported that they never visited websites related to health issues versus 23.2% (58/250) of smokers who were motivated to quit; this difference was maintained when demographic covariates were controlled for in a logistic regression analysis (*P*<.001, Wald=26.92, SE=0.22). Of those who visited health-related websites, there was a significant relationship between motivation to quit and frequency of visits.

Among those who had Internet access on their handheld devices (85.7%, 414/483), smokers who were motivated to quit were significantly more likely to have previously downloaded an app (91.6%, 206/225) than smokers who were not motivated to quit (79.9%, 151/189). There were no significant group differences when demographic variables were controlled.

Smokers who were motivated to quit were significantly more likely to have a Facebook account (82.4%, 206/250) than smokers who were not motivated to quit (70.4%, 176/250). There were no significant differences between groups when demographic variables were controlled for in a logistic regression analysis. Of those who had a Facebook account, there was a significant relationship between motivation to quit and the frequency of checking Facebook. Although none of the standardized residuals were equal to or greater than 1.96 or equal to or less than –1.96, the largest difference in percentages showed that smokers who were motivated to quit were more likely to report checking their Facebook pages more than once per day than smokers who were not motivated to quit (58.3%, 120/206 vs 48.3%, 85/176).

**Table 3:** Prevalence of technology use among smokers in the United Kingdom and differences in technology use by motivation to quit.

Variable			Total UK sample, n (%) n=500	Smokers not motivated to quit, n (%) n=250	Smokers motivated to quit, n (%) n=250	χ^2^ (*df*)	*P*
**Device ownership**							
	Own a mobile		479 (95.8)	233 (93.2)	246 (98.4)	8.40 (1)	.004
	Own a tablet		281 (56.2)	109 (43.6)	172 (68.8)	32.3 (1)	<.001
	Mobile is a smartphone		397 (82.9)	173 (74.2)	224 (91.1)	23.8 (1)	<.001
	Handheld device connects to the Internet		414 (85.7)	189 (80.4)	225 (90.7)	10.5 (1)	.001
**Texting**							
	Send text messages		462 (95.7)	223 (94.9)	239 (96.4)	0.6 (1)	.43
	Receive text messages		472 (97.7)	232 (98.7)	240 (96.8)	2.1 (1)	.15
	**Frequency of sending texts** ^a^					27.7 (5)	.001
		≤1 texts per month	41 (8.9)	27 (12.1)	14 (5.9)		
		2-4 texts per month	38 (8.2)	25 (11.2)	13 (5.4)		
		2-6 texts per week	87 (18.8)	48 (21.5)	39 (16.3)		
		1 text per day	30 (6.5)	17 (7.6)	13 (5.4)		
		2-9 texts per day	167 (36.1)	72 (32.3)	95 (39.7)		
		>10 texts per day^b^	99 (21.4)	34 (15.2)	65 (27.2)		
	**Frequency of receiving texts** ^c^					26.3 (5)	<.001
		≤1 texts per month	32 (6.8)	22 (9.5)	10 (4.2)		
		2-4 texts per month^b^	48 (10.2)	34 (14.7)	14 (5.8)		
		2-6 texts per week	84 (17.8)	44 (19.0)	40 (16.7)		
		1 text per day	35 (7.4)	16 (6.9)	19 (7.9)		
		2-9 texts per day	173 (36.7)	84 (36.2)	89 (37.1)		
		>10 texts per day^b^	100 (21.2)	32 (13.8)	68 (28.3)		
**Features of a handheld device used regularly**							
	Read email		338 (70.0)	139 (59.1)	199 (80.2)	25.6 (1)	<.001
	Get directions or use navigation (eg, GPS)		184 (38.1)	60 (25.5)	124 (50.0)	30.6 (1)	<.001
	Browse the Web		339 (70.2)	141 (60.0)	198 (79.8)	22.7 (1)	<.001
	Listen to music		257 (53.2)	88 (37.4)	169 (68.1)	45.7 (1)	<.001
	Take photos		300 (62.1)	118 (50.2)	182 (73.4)	27.5 (1)	<.001
	Check the news		286 (59.2)	111 (47.2)	175 (70.6)	27.2 (1)	<.001
	Record video		135 (28.0)	35 (14.9)	100 (40.3)	38.8 (1)	<.001
	Use apps (for any purpose)		321 (66.5)	129 (54.9)	192 (77.4)	27.5 (1)	<.001
	Search for information		333 (68.9)	131 (55.7)	202 (81.5)	37.2 (1)	<.001
	Play games by yourself		254 (52.6)	92 (39.1)	162 (65.3)	33.1 (1)	<.001
	Play games with other people		121 (25.1)	34 (14.5)	87 (35.1)	27.3 (1)	<.001
**Apps**							
	Previous app download^d^		357 (86.2)	151 (79.9)	206 (91.6)	11.8 (1)	.001
	**Longest used app for** ^d^					6.8 (4)	.14
		<1 day	36 (10.1)	13 (8.6)	23 (11.2)		
		≥1day but <1 week	56 (15.7)	21 (13.9)	35 (17.0)		
		≥1 week but <1 month	48 (13.4)	16 (10.6)	32 (15.5)		
		≥1 month but <1 year	95 (26.6)	50 (33.1)	45 (21.8)		
		≥1 year	122 (34.2)	51 (33.8)	71 (34.5)		
**Facebook**							
	Facebook account		382 (76.4)	176 (70.4)	206 (82.4)	10.00 (1)	.002
	**Frequency of checking** ^e^					13.0 (5)	.02
		Never	7 (1.8)	5 (2.8)	2 (1.0)		
		≤Once per month	22 (5.8)	16 (9.1)	6 (2.9)		
		2-4 times per month	26 (6.8)	12 (6.8)	14 (6.8)		
		2-6 times per week	43 (11.3)	25 (14.2)	18 (8.7)		
		Once per day	79 (20.7)	33 (18.8)	46 (22.3)		
		>Once per day	205 (53.7)	85 (48.3)	120 (58.3)		
**Visit health-related websites**			305 (61.0)	113 (45.2)	192 (76.8)	52.5 (1)	<.001
	**Frequency of visits** ^f^					15.7 (4)	.004
		≤Once per month	153 (50.2)	69 (61.1)	84 (43.8)		
		2-4 times per month	76 (24.9)	28 (24.8)	48 (25.0)		
		2-6 times per week	48 (15.7)	10 (8.8)	38 (19.8)		
		Once per day	18 (5.9)	6 (5.3)	12 (6.3)		
		>Once per day	10 (3.3)	0 (0)	10 (5.2)		

^a^ Of those who sent text messages (n*=*462).

^b^ Standardized residual ≥1.96 or ≤–1.96.

^c^ Of those who receive text messages (n*=*472).

^d^ Of those who could access the Internet on their handheld devices (n*=*414).

^e^ Of those with a Facebook account (n*=*382).

^f^ Of those who visit websites related to health issues (n*=*305).

### Previous use of Technology-Assisted Smoking Cessation Among US and UK Smokers and Differences by Motivation to Quit

#### Overview

Approximately one-quarter of smokers in the United States and United Kingdom reported that they previously used the Internet to quit smoking but the use of other technologies was low, ranging from 7.6% (38/500) for Twitter use in the United Kingdom to 15.2% (76/500) for smoking cessation app use in the United States (Table 4). Smokers in the United States were significantly more likely to have previously used Twitter (χ^2^_1_=5.9, *P*=.02), text messaging (χ^2^_1_=4.9, *P*=.03), and Facebook (χ^2^_1_=4.4, *P*=.04) to help them quit smoking than smokers in the United Kingdom.

There were significant differences in Internet-assisted cessation between smokers who were motivated to quit and smokers unmotivated to quit. Smokers who were motivated to quit were significantly more likely to have previously used each of the 5 assessed technologies to help them quit than smokers unmotivated to quit and these differences between groups were maintained when demographic covariates were controlled (Internet: *P*<.001, Wald=47.75, SE=0.01; text: *P*=.002, Wald=9.54, SE=0.01; quit smoking app: *P*<.001, Wald=33.72, SE*=*0.01; Twitter: *P*<.001, Wald=12.26, SE=0.01; and Facebook: *P*<.001, Wald=20.46, SE=0.01).

#### Future Intentions to Use Technology-Assisted Smoking Cessation Among US and UK Smokers and Differences by Motivation to Quit

Across both countries, the platforms with the greatest percentage of people endorsing that they would use it to quit smoking in the future were the Internet (46.7%, 467/1000) and apps (42.7%, 427/1000) (Table 5). Smokers in the United States were more likely to report that they would use text messages (χ^2^_1_=4.1, *P*=.04) and Twitter (χ^2^_1_=6.1, *P=*.01) to quit smoking in the future than smokers in the United Kingdom. Smokers who were motivated to quit were significantly more likely to say that they would use each of the 5 assessed technologies to help them quit smoking in the future than smokers unmotivated to quit, and these differences between groups were maintained when controlling for demographic covariates: future use of the Internet: *P<*.001, Wald=52.23, SE=0.01; text messages: *P=*.001, Wald=15.35, SE=0.01; quit smoking app: *P<*.001, Wald=54.40, SE=0.01; Twitter: *P*<.001, Wald=26.19, *SE*= 0.01; and Facebook: *P*<.001, Wald=27.21, SE=0.01.

**Table 4:** Previous use of technology-assisted smoking cessation among smokers in the United States and United Kingdom and differences by motivation to quit.

Previous use of technology to quit smoking		Total sample, n (%)	Unmotivated smokers, n (%)	Motivated smokers, n (%)	χ^2^_1_	*P*
**US Smokers**						
	Used the Internet (a website)	131 (26.2)	27 (10.8)	104 (41.6)	61.3	<.001
	Joined a quit smoking program that involved text messaging	66 (13.2)	12 (4.8)	54 (21.6)	30.8	<.001
	Used a quit smoking app on your phone	76 (15.2)	16 (6.4)	60 (24.0)	30.0	<.001
	Used Twitter to connect with other smokers who are trying to quit	61 (12.2)	13 (5.2)	48 (19.2)	22.9	<.001
	Used Facebook to connect with other smokers who are trying to quit	74 (14.8)	16 (6.4)	58 (23.2)	28.0	<.001
**UK Smokers**						
	Used the Internet (a website)	128 (25.6)	23 (9.2)	105 (42.0)	70.6	<.001
	Joined a quit smoking program that involved text messaging	44 (8.8)	6 (2.4)	38 (15.2)	25.5	<.001
	Used a quit smoking app on your phone	64 (12.8)	7 (2.8)	57 (22.8)	44.8	<.001
	Used Twitter to connect with other smokers who are trying to quit	38 (7.6)	3 (1.2)	35 (14.0)	29.2	<.001
	Used Facebook to connect with other smokers who are trying to quit	52 (10.4)	8 (3.2)	44 (17.6)	27.8	<.001

**Table 5:** Future intentions to use technology-assisted smoking cessation among smokers in the United States and United Kingdom and differences by motivation to quit.

Future intentions to use technology to quit		Total sample, n (%)	Unmotivated smokers, n (%)	Motivated smokers, n (%)	χ^2^_1_	*P*
**US Smokers**						
	Use the Internet (a website)	227 (45.4)	87 (34.8)	140 (56.0)	22.7	<.001
	Join a quit smoking program that involves text messaging	157 (31.4)	56 (22.4)	101 (40.4)	18.8	<.001
	Use a quit smoking app on your phone	217 (43.4)	90 (36.0)	127 (50.8)	11.2	<.001
	Use Twitter to connect with other smokers who are trying to quit	113 (22.6)	34 (13.6)	79 (31.6)	23.2	<.001
	Use Facebook to connect with other smokers who are trying to quit	152 (30.4)	53 (21.2)	99 (39.6)	20.0	<.001
**UK Smokers**						
	Use the Internet (a website)	240 (48.0)	89 (35.6)	151 (60.4)	30.8	<.001
	Join a quit smoking program that involves text messaging	128 (25.6)	43 (17.2)	85 (34.0)	18.5	<.001
	Use a quit smoking app on your phone	210 (42.0)	76 (30.4)	134 (53.6)	27.6	<.001
	Use Twitter to connect with other smokers who are trying to quit	82 (16.4)	22 (8.8)	60 (24.0)	21.1	<.001
	Use Facebook to connect with other smokers who are trying to quit	129 (25.8)	40 (16.0)	89 (35.6)	25.1	<.001

## Discussion

### Principal Findings

Both mHealth and eHealth interventions for smoking cessation are rapidly being developed and tested, but to our knowledge, there are no data on use of these technologies by smokers in general or whether use differs by motivation to quit. Knowing the types of technologies that smokers engage with can help intervention planners design interventions that target smokers more effectively and efficiently. The aims of our study were to (1) assess technology use among smokers in the United States and United Kingdom, (2) examine whether technology use differs between smokers who are motivated to quit and smokers who are not motivated to quit, (3) examine previous use of technology-based assisted smoking cessation, and (4) examine future intentions to use technology-based assisted smoking cessation. The advantages of mobile platforms include the ability to implement interventions in real time and access them any time and from any place, ability to tailor to user needs (eg, content, timing, and intensity), few barriers to participation, decreased time gap between treatment and behavior, low participant burden (particularly important for smokers who are less motivated to quit), ability to provide instant support, ability to provide feedback on goal setting and achievement, capability for integration with social networking, and scalability to large populations.

Among smokers in general, we found a high prevalence of mobile and smartphone ownership, sending and receiving texts, downloading and using apps, using Facebook, and visiting websites related to health. The use of these platforms, however, has outpaced the ability to gather scientific evidence regarding their effectiveness for smoking cessation. Although more than 400 smoking cessation mobile apps were available in 2013 [22], no fully powered randomized trials regarding their efficacy have been published yet (for pilot trials see [23,24]). Meta-analyses have shown that text messaging for smoking cessation is promising [13,25], but studies published to date have suffered from one or more methodological shortcomings, including lack of power, nonrandomization, short-term follow-up, lack of adequate control groups, lack of biochemical verification of cessation, and experimental designs that make it difficult to isolate the effects of text messaging on smoking cessation (eg, multicomponent interventions). With regard to Facebook, although 3 trials are currently underway, two do not assess smoking cessation as an outcome [26,27] and one is recruiting only smokers aged between 18 and 25 years [28]. Studies are also needed on dosage (eg, number of texts or app notifications needed for effectiveness), features that have the greatest potency for changing smoking behavior, theoretical mechanisms of action (“why” it works), and effectiveness for special populations (eg, those who are not motivated to quit).

The second aim of our study was to evaluate whether use of these platforms differs between smokers who are motivated to quit and those who are not motivated to quit. Although the prevalence of texting was high in both countries, US smokers who were not motivated to quit were less likely to text than US smokers who were motivated to quit. In both countries, smokers who were motivated to quit were supertexters and unmotivated smokers tended to text less frequently. More than a quarter of our total sample and approximately one-fifth of unmotivated smokers said they would be willing to use a text message program to quit smoking in the future. The ubiquity and frequency of text messaging among smokers in general and among unmotivated smokers specifically lends support to the idea that text messaging could also be used to motivate smokers to quit, perhaps serving as a way to keep cessation “on the radar,” titrating the number of text messages upward when and if the smoker becomes motivated to quit. Creative ways to keep unmotivated smokers engaged with this process should be explored. To date, text message interventions for smoking cessation have targeted only smokers who are ready and willing to set a quit date within 30 days.

Compared with unmotivated smokers, smokers who were motivated to quit tended to use their handheld devices more often to read email, get directions, browse the Web, listen to music, take photos/video, check the news, search for information, and play games. Intervention planners could capitalize on this information by examining the most prevalent features used by smokers and how they might reach smokers through these features. For example, more than 65% smokers who were motivated to quit reported that they played games on their handheld device. Thus, gaming principles could be incorporated into mobile cessation, possibly curbing smoking urges, providing distraction during times of temptation, and promoting self-efficacy for quitting. One preliminary study has shown that a prototype of an interactive game was engaging to smokers [29]. Our study extends this research by our finding that a large minority of unmotivated smokers play video games (39.2% in United Kingdom and 49.8% in United States), so involving gaming in promoting motivation to quit could be explored.

App downloads and length of app use differed by motivation group and by country. In the United Kingdom, motivated smokers were more likely to have downloaded an app than unmotivated smokers, but there were no differences in the length of time that apps were used. In the United States, there were no differences between motivation groups in the prevalence of downloading apps (both >88%). Research is needed regarding what features make a smoking cessation app “sticky” (ie, has staying power with the user) and designing apps that adhere to the human-centered design principles, including health literacy [30,31].

Although the vast majority of smokers had a Facebook account (UK: 76.4%; US: 87.0%), there were significant differences by motivation to quit for UK smokers, such that motivated smokers were more likely to have an account than unmotivated smokers. Regardless of motivation to quit, more than 72% checked Facebook once per day or more. There may be several advantages to delivering health behavior interventions through Facebook [32], such as the ability to reach participants while they interact in near real time, delivery of tailored content, ability to interface with apps, potential to influence perceptions of social norms [33], promote expansion of social networks beyond one’s own (which may have a high proportion of smokers), and incorporation of other media (eg, photos, video). Social network analytics can be automatically collected and used to measure delivery and use of behavior change techniques. Research is underway on using Facebook as a self-propagating delivery channel for smoking cessation by influencing behavior in local networks and facilitating diffusion (“viral spread”) between networks [26].

One striking finding is that unmotivated smokers were less likely to visit health-related websites (on their computer or handheld device) than were motivated smokers. Thus, these smokers need to be reached proactively through other media channels. This parallels the recommendations that were put forth before the advent of technology, that public health impact for smoking cessation could be achieved through proactive reach through existing infrastructures where smokers are located, such as primary care [34] and home health care [35], but also nontraditional settings such as beauty salons [36] and churches [37]. The current zeitgeist calls for finding and meeting smokers where they are located “electronically.” Previous proactive reach involved building relationships between smokers and providers to “hook” the unmotivated smokers. The next challenge for eHealth and mHealth is to find the electronic hook, one that will engage smokers regardless of their motivation to quit.

We assessed previous use of technology-assisted smoking cessation and found that more than 25% of smokers in both countries used the Internet to quit smoking. Other technology platforms had very low prevalence (Table 4), ranging from 12% to 15% in the United States and 7.6% to 12.8% in the United Kingdom. Thus, there is a discrepancy between the prevalence of these platforms (eg, >85% of sample use text messaging) and the prevalence of using the platform for smoking cessation (eg, 13.2% of US smokers and 8.8% of UK smokers joined a text message program for smoking cessation). This discrepancy could be reflective of a dearth of text message programs, smokers’ perception that text messaging would not be helpful, lack of marketing, or preference for human contact and support for smoking cessation. We also assessed future intentions to use technology-assisted smoking cessation and only two platforms (Internet and apps) exceeded 50% of the sample’s endorsement and that occurred only among motivated smokers. Among unmotivated smokers, Internet and apps had the highest endorsement at just over one-third in both countries.

### Limitations

One potential limitation of the current study is that participants were recruited online. There may be concern that this approach biases the sample to smokers who have Internet access. However, approximately 87% of the adult population of the United States and United Kingdom are Internet users [38,39], so our sample likely reflects the majority of smokers. In addition, we used a sampling strategy that ensured representativeness of smokers in each country. There may also be concern about the veracity of the data given that it was collected online. We checked for and eliminated duplicate IP addresses, random responders, and speeders and straight-liners to provide greater confidence in the data.

### Conclusions

Smoking cessation is an important public health goal, but the rate of cessation appears to have plateaued, meaning that new approaches are required. One possible approach is to devote greater efforts to understanding ways to target smokers who are not currently thinking of quitting smoking. This paper shows that although smokers who are not currently thinking of quitting make less use of technology than do smokers who are motivated to quit, sufficient numbers do use technology to make it worthwhile to develop these technologies designed to encourage unmotivated smokers to quit. Examining how health behavior change programs can capitalize on high rates of technology use is a public health priority, particularly because of the lack of disparities in the use of these technologies, relative low cost [40,41], and high potential for both customization and scalability.

## Abbreviations

FTND: Fagerström Test for Nicotine Dependence

## References

[1] Blackwell DL, Lucas JW, Clarke TC (2014). Summary health statistics for US adults: National Health Interview Survey, 2012. Vital Health Stat.

[2] (2013). Office for National Statistics.

[3] Borrelli Belinda (2010). Smoking cessation: next steps for special populations research and innovative treatments. J Consult Clin Psychol.

[4] Borrelli Belinda, Busch Andrew, Dunsiger Shira (2014). Cigarette smoking among adults with mobility impairments: a US population-based survey. Am J Public Health.

[5] (2009). Office for National Statistics.

[6] Centers for Disease Control and Prevention (2011). Quitting smoking among adults--United States, 2001-2010. MMWR Morb Mortal Wkly Rep.

[7] Fox S, Rainie L (2014). The Web at 25 in the US.

[8] Smith A (2011). Americans and Text Messaging.

[9] (2014). Pew Research Center.

[10] Kaplan Robert M, Stone Arthur A (2013). Bringing the laboratory and clinic to the community: mobile technologies for health promotion and disease prevention. Annu Rev Psychol.

[11] (2014). International Telecommunication Union.

[12] Duggan M (2013). Cell Phone Activities 2013.

[13] Whittaker R, McRobbie H, Bullen C, Borland R, Rodgers A, Gu Y (2012). Mobile phone-based interventions for smoking cessation. Cochrane Database Syst Rev.

[14] Free C, Whittaker R, Knight R, Abramsky T, Rodgers A, Roberts IG (2009). Txt2stop: a pilot randomised controlled trial of mobile phone-based smoking cessation support. Tob Control.

[15] Te Poel F, Bolman C, Reubsaet A, de Vries H (2009). Efficacy of a single computer-tailored e-mail for smoking cessation: results after 6 months. Health Educ Res.

[16] Brendryen H, Drozd F, Kraft P (2008). A digital smoking cessation program delivered through internet and cell phone without nicotine replacement (happy ending): randomized controlled trial. J Med Internet Res.

[17] Farago P (2013). Flurry Insights.

[18] Heatherton TF, Kozlowski LT, Frecker RC, Fagerström KO (1991). The Fagerström Test for Nicotine Dependence: a revision of the Fagerström Tolerance Questionnaire. Br J Addict.

[19] Baker TB, Piper ME, McCarthy DE, Bolt DM, Smith SS, Kim S, Colby S, Conti D, Giovino GA, Hatsukami D, Hyland A, Krishnan-Sarin S, Niaura R, Perkins KA, Toll BA (2007). Time to first cigarette in the morning as an index of ability to quit smoking: implications for nicotine dependence. Nicotine Tob Res.

[20] Rosen LD, Whaling K, Carrier LM, Cheever NA, Rokkum J (2013). The Media and Technology Usage and Attitudes Scale: an empirical investigation. Comput Human Behav.

[21] Segars AH, Grover V (1993). Re-examining perceived ease of use and usefulness: a confirmatory factor analysis. MIS Quarterly.

[22] Abroms LC, Lee Westmaas J, Bontemps-Jones J, Ramani R, Mellerson J (2013). A content analysis of popular smartphone apps for smoking cessation. Am J Prev Med.

[23] Bricker JB, Mull KE, Kientz JA, Vilardaga R, Mercer LD, Akioka KJ, Heffner JL (2014). Randomized, controlled pilot trial of a smartphone app for smoking cessation using acceptance and commitment therapy. Drug Alcohol Depend.

[24] Buller DB, Borland R, Bettinghaus EP, Shane JH, Zimmerman DE (2014). Randomized trial of a smartphone mobile application compared to text messaging to support smoking cessation. Telemed J E Health.

[25] Whittaker R, Borland R, Bullen C, Lin RB, McRobbie H, Rodgers A (2009). Mobile phone-based interventions for smoking cessation. Cochrane Database Syst Rev.

[26] Cobb NK, Jacobs MA, Saul J, Wileyto EP, Graham AL (2014). Diffusion of an evidence-based smoking cessation intervention through Facebook: a randomised controlled trial study protocol. BMJ Open.

[27] Sadasivam RS, Volz EM, Kinney RL, Rao SR, Houston TK (2013). Share2Quit: Web-based peer-driven referrals for smoking cessation. JMIR Res Protoc.

[28] Ramo DE, Rodriguez TM, Chavez K, Sommer MJ, Prochaska JJ (2014). Facebook recruitment of young adult smokers for a cessation trial: methods, metrics, and lessons learned. Internet Interv.

[29] Krebs P, Burkhalter JE, Snow B, Fiske J, Ostroff JS (2013). Development and alpha testing of QuitIT: an interactive video game to enhance skills for coping with smoking urges. JMIR Res Protoc.

[30] Broderick J, Devine T, Langhans E, Lemerise AJ, Lier S, Harris L (2014). Discussion Paper: Designing Health Literate Mobile Apps.

[31] De Vito Dabbs A, Myers BA, Mc Curry KR, Dunbar-Jacob J, Hawkins RP, Begey A, Dew MA (2009). User-centered design and interactive health technologies for patients. Comput Inform Nurs.

[32] Cobb NK, Graham AL, Byron MJ, Niaura RS, Abrams DB, Workshop Participants (2011). Online social networks and smoking cessation: a scientific research agenda. J Med Internet Res.

[33] Morris ME, Consolvo S, Munson S, Patrick K, Tsai J, Kramer AD (2011). Facebook for health: opportunities and challenges for driving behavior change. Proceedings of CHI EA ’11 Extended Abstracts on Human Factors in Computing Systems.

[34] Goldstein MG, Niaura R, Willey C, Kazura A, Rakowski W, DePue J, Park E (2003). An academic detailing intervention to disseminate physician-delivered smoking cessation counseling: smoking cessation outcomes of the Physicians Counseling Smokers Project. Prev Med.

[35] Borrelli B, Novak S, Hecht J, Emmons K, Papandonatos G, Abrams D (2005). Home health care nurses as a new channel for smoking cessation treatment: outcomes from project CARES (Community-nurse Assisted Research and Education on Smoking). Prev Med.

[36] Linnan LA, D'Angelo H, Harrington CB (2014). A literature synthesis of health promotion research in salons and barbershops. Am J Prev Med.

[37] Schorling JB, Roach J, Siegel M, Baturka N, Hunt DE, Guterbock TM, Stewart HL (1997). A trial of church-based smoking cessation interventions for rural African Americans. Prev Med.

[38] (2014). Office for National Statistics.

[39] (2014). Pew Research Center.

[40] Chen Y, Madan J, Welton N, Yahaya I, Aveyard P, Bauld L, Wang D, Fry-Smith A, Munafò MR (2012). Effectiveness and cost-effectiveness of computer and other electronic aids for smoking cessation: a systematic review and network meta-analysis. Health Technol Assess.

[41] Guerriero C, Cairns J, Roberts I, Rodgers A, Whittaker R, Free C (2013). The cost-effectiveness of smoking cessation support delivered by mobile phone text messaging: Txt2stop. Eur J Health Econ.

